# Quantitative multiparametric MRI as a non-invasive stratification tool in children and adolescents with autoimmune liver disease

**DOI:** 10.1038/s41598-021-94754-9

**Published:** 2021-07-27

**Authors:** Kamil Janowski, Elizabeth Shumbayawonda, Lin Cheng, Caitlin Langford, Andrea Dennis, Matt Kelly, Maciej Pronicki, Wieslawa Grajkowska, Malgorzata Wozniak, Piotr Pawliszak, Sylwia Chełstowska, Elzbieta Jurkiewicz, Rajarshi Banerjee, Piotr Socha

**Affiliations:** 1 Department of Gastroenterology, Hepatology, Nutritional Disorders and Pediatrics, The Children’s Memorial Health Institute, Warsaw, Poland; 2 Perspectum Ltd., Gemini One, 5520 John Smith Drive, Oxford, UK; 3 Department of Pathology, The Children’s Memorial Health Institute, Warsaw, Poland; 4 Department of Diagnostic Imaging, The Children’s Memorial Health Institute, Warsaw, Poland

**Keywords:** Biliary tract, Hepatology, Autoimmune hepatitis, Diagnostic markers

## Abstract

Autoimmune hepatitis (AIH) and autoimmune sclerosing cholangitis (ASC) are two very closely related autoimmune liver diseases with overlapping clinical features and similar management strategies. The purpose of this study was to assess the utility of quantitative imaging markers to distinguish ASC from AIH in paediatrics. 66 participants (N = 52 AIH, N = 14 ASC) aged 14.4 ± 3.3 years scheduled to undergo routine biopsy and baseline serum liver biochemistry testing were invited to undergo MRI (non-contrast abdominal MRI and 3D fast spin-echo MRCP). Multiparametric MRI was used to measure fibro-inflammation with corrected T1 (cT1), while the biliary tree was modelled   using quantitative MRCP (MRCP +). Mann–Whitney U tests were performed to compare liver function tests with imaging markers between patient groups (ASC vs AIH). Receiver operating characteristic curves and stepwise logistic regressions were used to identify the best combination of markers to discriminate between ASC and AIH. Correlations between liver function tests and imaging markers were performed using Spearman’s rank correlation. cT1 was significantly correlated with liver function tests (range 0.33 ≤ R ≤ 56, *p* < 0.05), as well as with fibrosis, lobular and portal inflammation (range 0.31 ≤ R ≤ 42, *p* < 0.05). 19 MRCP + metrics correlated significantly with liver function tests (range 0.29 ≤ R ≤ 0.43, *p* < 0.05). GGT and MRCP + metrics were significantly higher in ASC compared to those with AIH. The best multivariable model for distinguishing ASC from AIH included total number of ducts and the sum of relative severity of both strictures and dilatations AUC: 0.91 (95% CI 0.78–1). Quantitative MRCP metrics are a good discriminator of ASC from AIH.

## Introduction

Autoimmune hepatitis (AIH) is a complex condition that presents in both acute and chronic forms in patients of all ages^1^. It remains a diagnosis of exclusion since there is no disease-specific test and one third of patients present with advanced liver disease. Autoimmune sclerosing cholangitis (ASC), which is arguably the most frequent form of sclerosing cholangitis in children^2^, was originally described by Gregorio et al.^3^ and is a form of sclerosing cholangitis with strong autoimmune features overlapping with AIH. ASC is generally diagnosed (using biopsy and consecutive histopathology) and currently managed in the same way as AIH, but usually in combination with ursodeoxycholic acid (UDCA)^2^. Moreover, although AIH patients generally have a higher median inflammatory activity index, and ASC has a higher frequency of cholangitis and associated inflammatory bowel disease (IBD) when compared to AIH, overall clinical presentation, blood markers, histology (including antibodies and interface hepatitis) are similar in the two conditions^4,5^. Due to the similarities between AIH and ASC, there are multiple open issues surrounding diagnosis and monitoring of ASC^2^ especially as both indications have very similar presentation and monitoring. Therefore, there is a need for the introduction of other markers, to form criteria which can be used to support diagnosis and facilitate improved patient monitoring^3,6^.

Non-invasive techniques have a unique role in disease characterisation^7–10^, assessment of treatment response^11^, clinical outcome prediction^12,13^, and patient monitoring^14^. Magnetic resonance cholangiopancreatography (MRCP) is an MRI technique that characterises the health of the biliary tree and has a recognised role as a surrogate marker for diagnosis and disease monitoring. Interpretation of MRCP, however, currently relies on qualitative evaluation and is therefore prone to inter-observer variability^15,16^, limiting its potential to correctly identify disease features and detect change over time*.* Although such disagreements in interpretation are less likely to happen in research studies which make use of expert readers, in clinical practice and in the interpretation of MRCP images from children these disagreements have a higher potential to occur. Quantitative MRCP (MRCP +), is a novel image processing tool that provides quantitative metrics derived from 3D MRCP images obtained in a relatively short imaging time^17,18^. More specifically, MRCP + uses a standardized imaging protocol and image processing software to produce a 3D model of the biliary tree which can be used to both visualise the biliary ducts, as well as to provide quantitative measures for the direct assessment of ductal anatomy^18^. These metrics, which enable objective evaluation of aspects such as the biliary tree volume, duct length and diameter as well as the presence of strictures and dilatations, have been shown to be repeatable and reproducible^18^ as well as having early utility in the monitoring of paediatric autoimmune liver disease^17^.

In addition to evaluating the differences in the biliary tree characteristics between ASC and AIH using MRCP +, characterising the fibrosis and inflammation within the liver parenchyma and the changes brought on by treatment can also help in monitoring disease progression. Iron corrected T1 (cT1), a multiparametric MRI (mpMRI) measure of fibrosis and inflammation (fibro-inflammation)^7–11,14,19,20^, has shown utility in the rapid assessment of treatment response in liver disease^11^ as well as the prediction of future loss of biochemical remission and the occurrence of flares in AIH^13^. cT1 has also been shown to identify children and adolescents with normal biochemistry but having (histologically confirmed) active sub-clinical disease^14^, and to indicate the presence of radiologic portal hypertension in chronic progressive paediatric autoimmune liver diseases^12^.

Previous work done by Gilligan et al.^17^ using the basic MRCP + metrics showed excellent utility to stratify between ASC and AIH. The aim of this study was to assess the diagnostic performance and utility of using quantitative MRI metrics, and more specifically advanced MRCP + metrics, to distinguish ASC from AIH in children and adolescents.

## Materials and methods

### Patient recruitment and assignment of clinical diagnosis

Children aged 6–18 with biopsy confirmed or suspected AIH or ASC under care of hepatologists at the Children’s Memorial Health Institute in Warsaw (IPCZD) were invited to have a research non-contrast MRI scan alongside their clinical history assessment, examination, and serum liver biochemistries. 66 participants were included in the analysis in this study: 52 with AIH and 14 with ASC (Table 1). AIH was diagnosed following medical guidelines using liver histology and assessing for the absence of radiological or histopathological evidence of cholangiopathy, while ASC was diagnosed as AIH with radiological or histopathological features of PSC^21–23^. In this study, the MRI scan was performed in 3 ± 6 days before the liver biopsy was performed.

**Table 1:** Patient demographics and characteristics.

	AIH (N = 52)	ASC (N = 14)	*p* value
**Patient demographics**			
Female (%)	23 (60.5%)	5 (45.5%)	0.37
Age (years)	14.4 ± 3.4	14.2 ± 3.0	0.83
BMI (kg/m^2^)	20.4 ± 4.1	21.4 ± 3.8	0.46
Time from diagnosis to MRI (years)	2.2 ± 2.1	2.9 ± 3.7	0.71
**Liver biochemistry**			
Total Bilirubin (mg/dL)	1.1 ± 1.7	0.8 ± 0.4	0.47
ALT (IU/L)	181.4 ± 347.9	103.8 ± 103.9	0.39
AST (IU/L)	168.4 ± 355.7	104.4 ± 132.9	0.26
GGT (IU/L)	73.3 ± 128.9	224.8 ± 399.9	**0.024**
IgG (g/L)	14.0 ± 5.2	14.6 ± 4.9	0.61
Albumin (g/L)	43.1 ± 5.0	42.9 ± 3.0	0.47
Gamma globulins (%)	18.9 ± 6.4	16.6 ± 4.5	0.28
**Histology scores**			
Fibrosis			
Score 0	6	2	
Score 1	10	0	
Score 2	8	2	
Score 3	11	4	
Score 4	7	1	
Score 5	8	3	
Score 6	2	2	
Portal inflammation			
Score 0	6	1	
Score 1	17	6	
Score 2	19	6	
Score 3	10	1	
Lobular inflammation			
Score 0	17	5	
Score 1	28	5	
Score 2	3	3	
Score 3	4	1	

The different section headings, significant *p* values, and patient groups in the column headers have bold font. They have been put in bold text to aid in the interpretation of the data presented.

This prospective observational study (NCT03198104) was sponsored by the Eureka Eurostars 2 Grant (E!10124) and received ethical approval (11/KBE/2016) from the Komisja Bioetyczna przy Instytucie “Pomnik-Centrum Zdrowia Dziecka” (Ethic Committee at the Children's Memorial Health Institute in Warsaw) in Poland. Principals of Good Clinical Practice and those of the 1975 Declaration of Helsinki were observed throughout the study. All participants and their parents, guardians or legal caregivers gave informed consent to take part in the study.

### Clinical, laboratory and histology data

Serum liver biochemistries were obtained as part of routine clinical care at the time of the research MRI. For each patient liver biochemistry data (namely alanine aminotransferase (ALT, IU/L), aspartate transaminase (AST, IU/L), gamma-glutamyl transferase (GGT, IU/L), total bilirubin (mg/dL), immunoglobulin G (IgG, g/L), and gamma globulins (%)) were recorded alongside demographic data and the time between diagnosis and MRI imaging (disease duration) (Table 1). Percutaneous liver biopsy samples were assessed histopathologically for Ishak fibrosis (scale 0–6) as well as lobular and portal inflammation (scale 0–2) by two experienced liver pathologists.

### Image acquisition and post‑processing

MRCP images were obtained using 3D multi-shot fast/turbo spin echo acquisitions, with very long echo train lengths and short echo spacing, to generate heavily T2-weighted three-dimensional volumetric images. Seventy-two contiguous slices were acquired with a field of view of 400 × 400, an acquisition matrix of 258 × 320, and a reconstruction matrix of 320 × 320, resulting in a voxel resolution of 1.25 × 1.1 × 1.25 mm for all scans. Data was acquired with respiratory gating (using navigator tracking) and during the expiration phase, so that the repetition time (TR) varied with breathing rate. Fat suppression techniques were used to suppress signal from fat, and parallel imaging techniques to reduce scanning time. Post-processing software, MRCP + ™ (Perspectum Ltd., United Kingdom), was then used to extract and process the maximum intensity projection of the acquired MRCP data to derive quantitative metrics and create a colour-coded 3D model of the biliary tree showing the variation in diameter along each duct as described by Goldfinger et al^18^.

**Figure 1:**
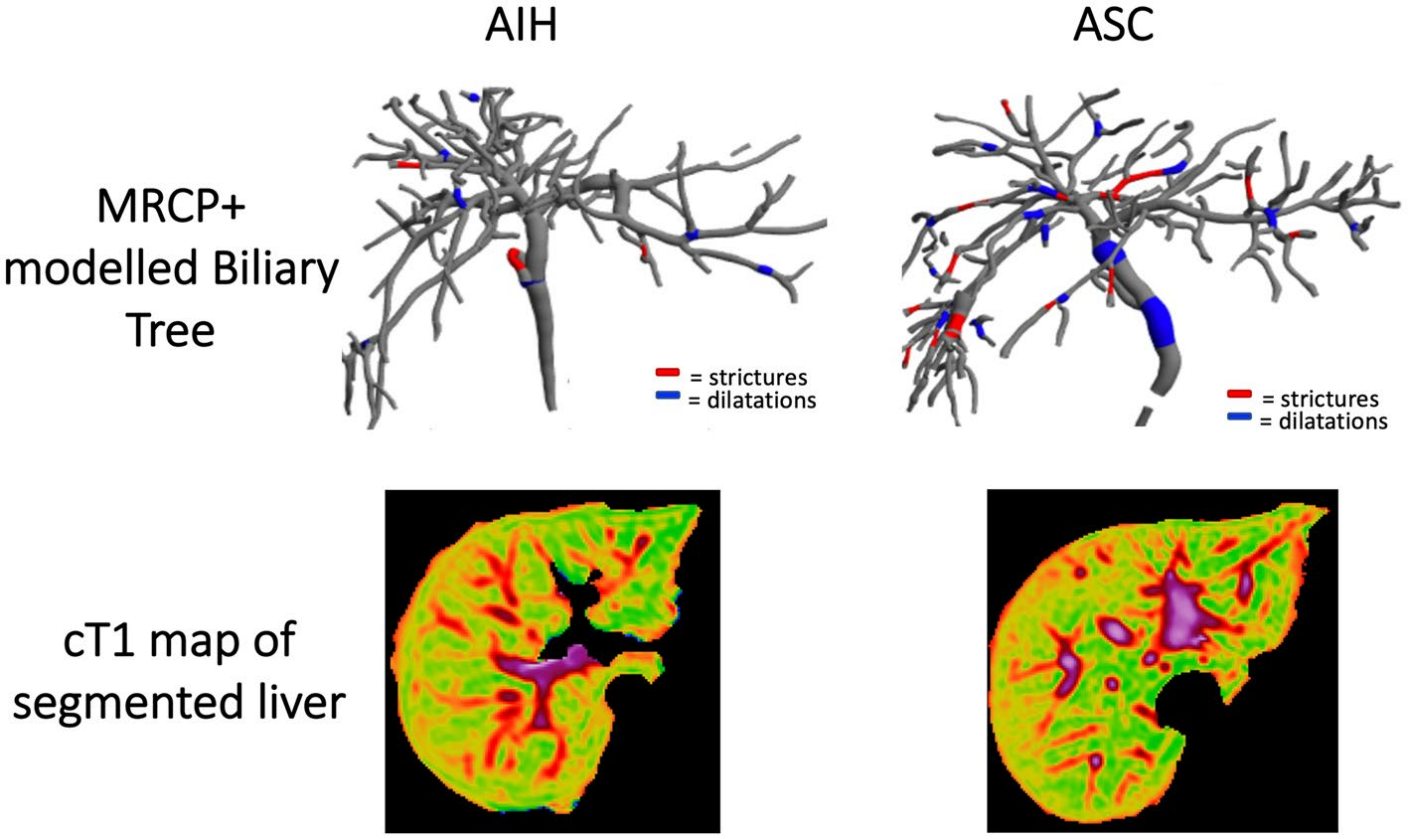
Representative biliary tree models and cT1 maps from an AIH patient (left) and an ASC patient (right). For the ASC patients, the MRCP + model shows the presence of multiple strictures (red) and dilatations (blue).

In the same scanning session, non-contrast T1, T2*, and proton density fat fraction (PDFF) mapping was acquired using the LiverMultiScan® protocol (Perspectum Ltd., United Kingdom) with 4 transverse slices were captured through the centre of the liver through the porta hepatis in a similar manner as described in detail by Bachtiar et al.^24^. During image analysis using LiverMultiScan® circular regions of interest were placed on 4 slices on the transverse T2* and PDFF maps, while cT1 maps of the liver were delineated into whole liver segmentation maps using a semi-automatic method^24^. All scans were performed at the IPCZD on 1.5 T Siemens Avanto systems (Siemens Healthineers, Germany). All images were analysed by trained analysts blinded to the clinical data. Figure 1 shows an illustration of the resultant 3D model quantitative model of the biliary tree derived using MRCP + as well as the cT1 map for both an AIH and ASC patient.

### Statistical analysis

Descriptive statistics were used to summarise baseline participant characteristics with the normality of each metric assessed using Shapiro–Wilk test and visual inspection using histograms. Continuous normally-distributed variables were reported as mean and standard deviation (SD), continuous non-normally distributed variables were reported as median and interquartile range (IQR), categorical variables were reported as frequency and percentage. Disease duration was calculated as the time (days) between diagnosis biopsy and MRI scan.

To assess the differences between AIH and ASC, Mann–Whitney U tests with continuity correction were performed to compare continuous variables (biochemical serum markers and imaging metrics) between patient cohorts. As MRCP + provides 58 metrics characterising the whole biliary tree as well as ducts, strictures and dilatations, all metrics were correlated with biochemical serum markers and histology markers (fibrosis and inflammation). A list of all the metrics obtained using MRCP + are presented in supplementary Table 1. Those which did not correlate with either biochemical serum or histology markers were discarded. Following this, univariate logistic regression models were fitted to assess the diagnostic performance of individual imaging predictors (all biochemical serum markers, mpMRI metrics, and the remaining MRCP + metrics that correlated with biochemical serum markers and histology markers). Receiver operating characteristic (ROC) curves were generated and area under the ROC curve (AUR) as well as its 95% CI was estimated. Sensitivity, and specificity were calculated for each marker (biochemical serum markers and quantitative MRCP metric) from the best cut-off determined using the Youden’s Index.

Using only the markers (both MRCP + metrics and biochemical serum markers) with high individual performance (AUC > 0.7), stepwise logistic regressions were then used to select a reduced number of metrics to build the best performing logistic regression model. The best performing multivariable model was chosen based on the lowest Akaike Information Criterion. Finally, a multivariate logistic regression model was fitted to the best combination of MRCP + metrics for discriminating the two patient cohorts.

All statistical analyses were performed using R version 3.5.3 (R Core Team, Vienna, Austria), and values of *p* < 0.05 were considered statistically significant.

## Results

### Patient demographics

66 participants were invited to undergo research MRI (mpMRI and MRCP +) alongside all standard clinical procedures were included into our study. Case deletion of 19 entries with missing data (10 missing clinical or laboratory data, while 9 had MRCP scans that could not be post-processed due to motion artefact) was performed, and thus 47 patients were included in this study. Detailed demographic data of all participants recruited into the study is presented in Table 1.

### Associations between imaging biomarkers and serum markers of AIH activity

The value of imaging biomarkers and their relationship to traditional serum measurements of liver biochemistry was assessed in the 47 AILD patients. Positive correlations between cT1 and ALT (R = 0.52, *p* < 0.0001), AST (R = 0.56, *p* < 0.0001), GGT (R = 0.5, *p* < 0.0001), total bilirubin (R = 0.46, *p* = 0.001), IgG (R = 0.34, *p* = 0.015). Associations between cT1 and histology, showed that cT1 significantly correlated with fibrosis (R = 0.42, *p* = 0.002) as well as lobular (R = 0.31, *p* = 0.03) and portal (R = 0.41, *p* = 0.003) inflammation.

MRCP + metrics quantifying ductal characteristics (the percentage of ducts with median range (1–3 mm), the percentage of ducts with median range (3–5 mm), the percentage of ducts with diameter range 1–3 mm, the percentage of ducts with diameter range 3–5 mm) and mean absolute severity of candidate strictures (range − 0.34 < R < 0.35, *p* < 0.05) correlated significantly with fibrosis, while the percentage of ducts with diameter range < 1 mm (R = 0.31, *p* = 0.029) correlated with lobular inflammation. Most MRCP + metrics correlated significantly with GGT (range 0.29 < R < 0.43, *p* < 0.05), while some metrics correlated with AST (range 0.31 < R < 0.37, *p* < 0.05), ALT (range 0.29 < R < 0.42, *p* < 0.05), IgG (range 0.29 < R < 0.35, *p* < 0.05) and total bilirubin (range 0.3 < R < 0.32, *p* < 0.05). Thus, of the 58 metrics considered, 19 metrics correlated significantly with biochemical serum markers (supplementary table 2) and histology markers of fibrosis and inflammation (supplementary table 3).

### Differences between AIH and ASC biliary structure

Apart from GGT that was significantly higher (*p* = 0.02) in ASC compared to those with AIH, no other significant differences were found in the liver biochemistry markers between the 2 groups (Table 1). In addition to this, mpMRI markers (cT1 and liver fat) were also not found to be significantly different between the two groups (*p* > 0.05) (Table 2). Nevertheless, 14/19 MRCP + metrics were found to be significantly different between those with AIH and ASC (Table 2).

**Table 2:** Quantitative MRCP and mpMRI data per AILD cohort (AIH vs. ASC)—report Median and IQRs.

	AIH (N = 35)	ASC (N = 14)	*p*
**mpMR**I			
cT1	780 ± 76.0	801.5 ± 71.7	*0.41*
PDFF	3.8 ± 11.1	1.7 ± 0.9	*0.54*
**Quantitative MRCP metrics**			
Percentage of the ducts with median diameter ranging 1–3 mm	87.5 (8.9)	87.8 (8.3)	*0.92*
Percentage of the ducts with median diameter ranging 3–5 mm	11.5 ± 7.8	11.1 ± 7.9	*0.89*
Percentage of the ducts with median diameter ranging 5–7 mm	0.0	0.3 ± 0.5	**< *0.001***
Percentage points along the bile duct centreline with diameter < 1 mm	0.2 ± 0.2	0.1 ± 0.1	*0.43*
Percentage points along the bile duct centreline with diameter ranging 1–3 mm	82.9 ± 8.6	82.9 ± 9.5	*0.99*
Percentage points along the bile duct centreline with diameter ranging 3–5 mm	15.6 ± 7.3	15.4 ± 8.4	*0.95*
Percentage points along the bile duct centreline with diameter ranging 7–9 mm	± 0.2	0.3 ± 0.5	***0.018***
Percentage points along the bile duct centreline with diameter < 9 mm	0.0	± 0.2	***0.016***
Total number of strictures	3.3 ± 3.1	13.2 ± 15.7	**< *0.001***
Sum of absolute severity of strictures	4.3 ± 4.7	18.8 ± 23.9	**< *0.001***
Sum of relative severity of strictures	137.5 ± 130.7	551.2 ± 649.4	**< *0.001***
Total number of dilatations	6.5 ± 6.3	26.4 ± 33.3	**< *0.001***
Total length of dilatations (mm)	46.0 ± 44.5	149.2 ± 176.5	***0.002***
Maximum dilatation diameter	5.1 ± 1.3	6.9 ± 2.2	***0.001***
Sum of relative severity of candidate dilatations	442.5 ± 440.0	1633.2 ± 2166.6	***0.002***
Number of ducts with strictures	2.9 ± 2.4	11.2 ± 12.4	**< *0.001***
Number of ducts with dilatations	5.6 ± 4.9	20.8 ± 24.1	**< *0.001***
Number of ducts with strictures/dilatations	6.9 ± 5.3	24.5 ± 26.0	**< *0.001***
Total length of ducts with abnormalities (strictures and dilatations) (mm)	72.5 ± 62.7	251.9 ± 285.9	**< *0.001***

The different section headings, significant *p* values, and patient groups in the column headers have bold font. They have been put in bold text to aid in the interpretation of the data presented.

### Predictive capability of quantitative imaging

**Figure 2:**
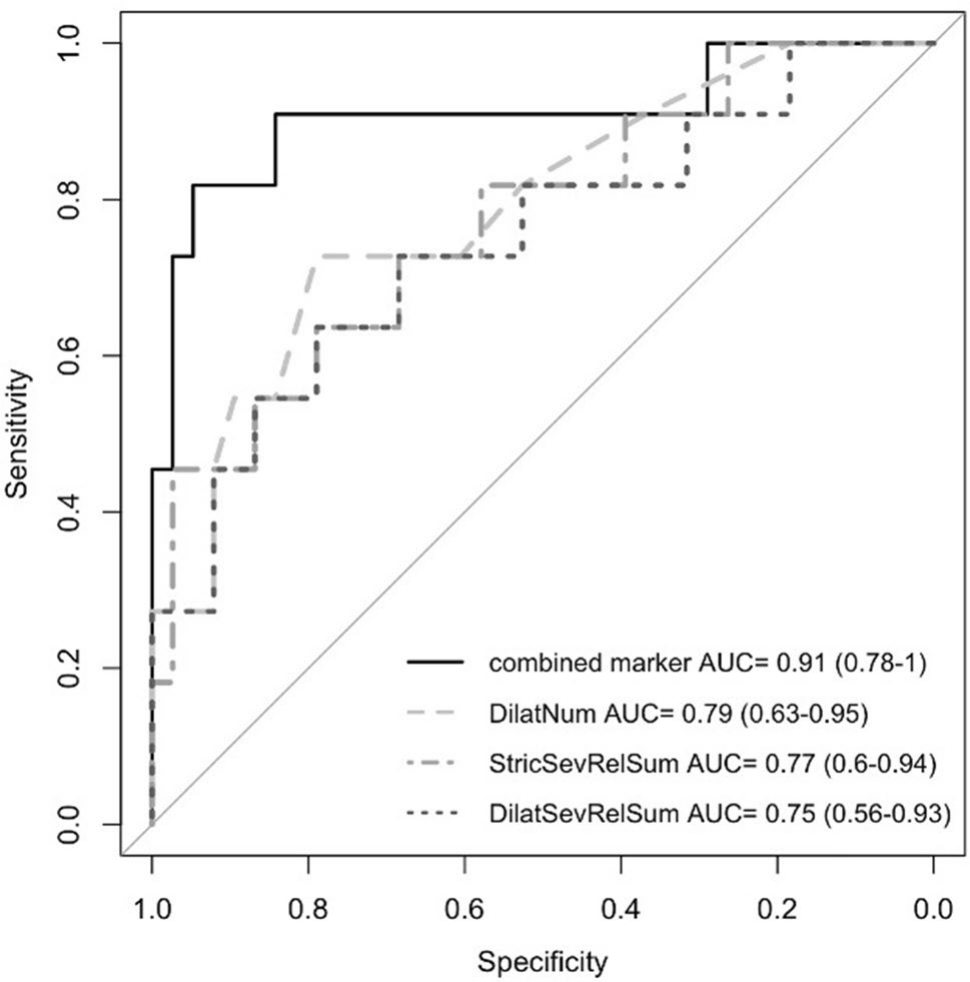
ROC curve of the best performing multivariable logistic regression model for discrimination of ASC from AIH (DilatNum—Total number of dilatations, StricSevRelSum—Sum of relative severity of strictures, and DilatSevRelSum—Sum of relative severity of candidate dilatations).

ROC curve analyses were performed on all biomarkers to distinguish between those with AIH and ASC, the results of which are summarised in Table 3. Of the biochemical serum markers, GGT was found to have good performance (AUC > 0.7) with AUC: 0.73 (95% CI 0.57–0.88). cT1, similar to ALT and AST, had moderate discriminatory performance between AIH and ASC (AUC < 0.7) and had AUC: 0.59 (95% CI 0.4–0.79). 11/19 MRCP + metrics were found to be good discriminators between AIH and ASC (AUC > 0.7). The MRCP + metrics with the highest individual capability for distinguishing ASC from AIH were the number of ducts with strictures/dilatations (AUC: 0.81, 95% CI 0.65–0.96), number of ducts with dilatations (AUC: 0.79, 95% CI 0.64–0.94), total number of ducts (AUC: 0.79, 95% CI 0.63–0.95), and number of ducts with strictures (AUC: 0.78, 95% CI 0.61–0.95) (Table 3). Stepwise logistic regression were performed to obtain the best combination of markers (all markers shown in Table 3 with AUC > 0.7) to discriminate ASC from AIH. Results showed that the combination of total number of ducts (OR 5.76; 95% CI 1.75–39.08), the sum of relative severity of strictures (OR 1.02; 95% CI 1.00–1.05), and the sum of relative severity of dilatations (OR 0.97; 95% CI 0.94–0.99) achieved an AUC: 0.91 (95% CI 0.78–1) (Fig. 2).

**Table 3:** Assessment of the diagnostic performance of clinical and imaging parameters for discriminating ASC from AIH using receiver operating characteristic (ROC) curve analyses.

	AUC	95% CI	Cut-off	Sensitivity	Specificity
**Liver biochemistry**					
ALT	0.59	0.4–0.78	47.50	0.73	0.63
AST	0.61	0.43–0.79	32.50	0.73	0.55
GGT	0.73	0.57–0.88	22.50	0.91	0.50
Total bilirubin	0.45	0.27–0.63	1.61	1.00	0.16
IgG	0.55	0.35–0.75	13.00	0.64	0.55
Gamma globulins	0.61	0.4–0.82	15.25	0.55	0.74
**mpMRI**					
PDFF	0.45	0.22–0.67	1.60	0.55	0.61
cT1	0.59	0.4–0.79	801	0.71	0.46
**Biliary tree parameters**					
Percentage of the ducts with median diameter ranging 1–3 mm	0.5	0.30–0.70	92.32	0.73	0.42
Percentage of the ducts with median diameter ranging 3–5 mm	0.52	0.32–0.72	16.43	0.82	0.32
Percentage of the ducts with median diameter ranging 5–7 mm	0.64	0.5–0.77	0.43	0.27	1.00
Percentage points along the bile duct centreline with diameter < 1 mm	0.52	0.35–0.69	0.014	0.64	0.53
Percentage points along the bile duct centreline with diameter ranging 1–3 mm	0.50	0.29–0.71	78.17	0.36	0.74
Percentage points along the bile duct centreline with diameter ranging 3–5 mm	0.51	0.3–0.72	11.48	0.45	0.74
Percentage points along the bile duct centreline with diameter ranging 7–9 mm	0.66	0.49–0.83	0.02	0.45	0.87
Percentage points along the bile duct centreline with diameter < 9 mm	0.68	0.53–0.83	0.01	0.36	1.00
Total number of strictures	0.77	0.6–0.94	10.00	0.45	0.97
Sum of absolute severity of strictures	0.77	0.59–0.95	5.20	0.73	0.76
Sum of relative severity of strictures	0.77	0.6–0.94	439.82	0.45	0.97
Total number of dilatations	0.79	0.63–0.95	8.50	0.73	0.79
Total length of dilatations (mm)	0.72	0.52–0.92	45.67	0.73	0.74
Maximum dilatation diameter	0.76	0.58–0.94	5.11	0.91	0.55
Sum of relative severity of candidate dilatations	0.75	0.56–0.93	570.04	0.64	0.79
Number of ducts with strictures	0.78	0.61–0.95	8.00	0.45	0.97
Number of ducts with dilatations	0.79	0.64–0.94	10.50	0.55	0.89
Number of ducts with strictures/dilatations	0.81	0.65–0.96	6.50	0.91	0.58
Total length of ducts with abnormalities (strictures and dilatations) (mm)	0.76	0.59–0.94	140.54	0.55	0.87

## Discussion

In this study we report the diagnostic utility of quantitative MRCP in the discrimination of two very closely related autoimmune liver diseases AIH and ASC. The findings from this study strengthen the existing literature highlighting the utility of biliary tree metrics derived from quantitative MRCP to provide good discrimination between types of autoimmune liver disease (AILD). Moreover, results also showed that although cT1 was not significantly different between those with AIH and those ASC, its utility lies more in the characterization and monitoring of parenchymal disease than in the stratification of closely related AILDs.

MRCP already plays a significant role in the assessment, diagnosis, and monitoring of AILD with biliary involvement, however, multiple limitations associated with the subjective nature of the interpretation of MRCP^25,26^ mean that this technique, in its current form, cannot be used to provide biomarkers which can predict key clinical outcomes. For instance, although expert trained radiologists can accurately diagnose PSC using MRCP, there is high inter-observer variability, especially in the detection of more atypical findings in PSC^15,16^. Quantitative MRCP is a novel technique with good repeatability and reproducibility across multiple scanner platforms^18^, and recent refinement of the technology to allow for better segmentation of the biliary tree^27^ resulted in a decrease in the scan rejection rate due to motion artefact of 14% (reduced from 30% as reported previously^17^).

MRCP + provides a quantitative overview of the worsening of the biliary tree, and thus provide useful information that can be used to support diagnosis and facilitate improved patient monitoring^6^. As ASC and AIH are both closely related in parenchymal disease progression and serum biochemistry, the biliary tree of patients with ASC is typically distinguished from those with AIH by its “beaded” appearance in MRCP, resulting from the presence of biliary strictures and dilations^28^. Thus, the emergence of the relative severity of both strictures and dilatations as significant metrics in the diagnostic model highlights the inherent divergence between the two AILDs. Moreover, dilatations are known to increase with time in sclerosing cholangitis and thus, the emergence of the total number of dilatations as a significant metric (AUC: 0.79) further highlights the structural difference that exist between those with AIH and those with ASC. By combining the number of dilatations with the relative severity of both the strictures and dilatations, good diagnostic predictive capability (AUC 0.91) to discriminate between ASC and AIH can be obtained. Thus, this shows that quantitative characterisation of the biliary tree has a role to play in understanding the differences between ASC and AIH. This is especially so as the derived metrics can potentially be used to further understanding the components of autoimmune attack directed towards both the bile ducts and the liver parenchyma seen in ASC^3,6^. As there is no medical treatment that has been shown to significantly alter its progression, sclerosing cholangitis typically follows a worsening course^29^ and thus, this information could also 1 day be used to inform physician decision and thus positively improve patient monitoring.

While the results described above are promising and relevant within the AILD space, there are some limitations to our study. Firstly, we had a relatively small cohort of ASC patients. Nevertheless, as multiple studies have shown the prevalence of ASC in AIH to range from 1.7 to 33.0%^30^ the size of our cohort may be considered representative of current clinical frequencies. Secondly, no comparisons between qualitative and quantitative MRCP were performed, and thus, the differences between the two forms of interpretation were not evaluated. Patients with primary sclerosing cholangitis (PSC) were also not included in the cohort recruited into this study as it was not part of standard of care at IPCZD to routinely biopsy this patient group. This exclusion is a limitation to our study as investigation into the similarities/differences between the sclerosing cholangitis types was not performed. Therefore, studies looking at the utility of these metrics to differentiate between sclerosing cholangitis types are needed so as to better understand the sensitivity of this new technique. Lastly, this was a cross-sectional study, and evaluation of the utility of these markers to both monitor disease progression/regression or their ability to predict clinical outcomes was not performed. Future studies looking at longitudinal assessment will yield a better understanding of the changes associated with these metrics, and thus will reveal the impact these metrics have on monitoring of disease progression over time, the sensitivity of the metrics to change and their associations with important clinical outcomes.

In conclusion, quantitative MRCP provides numerous imaging biomarkers that can be used to evaluate the biliary tree in a manner that can successfully highlight the differences between patients with ASC and those with AIH. Moreover, by combining MRCP + metrics that quantify the relative severity of both strictures and dilatations (characteristic of sclerosing cholangitis) in the entire biliary tree with the total number of dilatations in the biliary tree, good discrimination between ASC and AIH was achieved. Thus, the further strengthening the utility of using quantitative MRCP to provide clinically useful information which can positively aid patient management.

## Supplementary Information

Supplementary Tables.

## Abbreviations

AIH: Autoimmune hepatitis
ASC: Autoimmune sclerosing cholangitis
UDCA: Ursodeoxycholic acid
IBD: Inflammatory bowel disease
MRCP: Magnetic resonance cholangiopancreatography
MRCP +: Quantitative MRCP
mpMRI: Multiparametric MRI
fibro-inflammation: Fibrosis and inflammation
IPCZD: Children’s Memorial Health Institute in Warsaw
ALT: Alanine aminotransferase
AST: Aspartate transaminase
GGT: Gamma-glutamyl transferase
IgG: Immunoglobulin G
PDFF: Proton density fat fraction
cT1: Corrected T1
SD: Standard deviation
IQR: Interquartile range
ROC: Receiver operating characteristic
AUC: Area under the ROC curve
AILD: Autoimmune liver disease
PSC: Primary sclerosing cholangitis
