# Supplementary material for: Quantitative multiparametric MRI as a non-invasive stratification tool in children and adolescents with autoimmune liver disease
Source: Sci Rep. 2021 Jul 27;11:15261. doi: 10.1038/s41598-021-94754-9 (PMC8316432; doi:10.1038/s41598-021-94754-9)
Supplement: Supplementary file 1 — Supplementary Tables. [file 41598_2021_94754_MOESM1_ESM.docx]

**Quantitative multiparametric MRI as a non-invasive stratification tool in children and adolescents with autoimmune liver disease**

**Supplimentary table 1: Summary of the metrics obtained from MRCP+ annd how these metrcis are grouped.**

**Supplimentary table 2: Correlations between imaging metrics and liver biochemistry.**

**Supplementary table 3: Correlations between imaging metrics, liver biochemistry and histology**

Supplementary table 1: Summary of the metrics obtained from MRCP+ annd how these metrcis are grouped.

| **Group** | **Metrics** |
| --- | --- |
| Number of ducts affected by a pathology | Total number of modelled ducts |
|  | Total number of candidate strictures |
|  | Total number of candidate dilatations |
|  | Number of ducts containing one or more strictures |
|  | Number of ducts containing one or more dilatations |
|  | Number of ducts containing one or more strictures OR dilatations |
|  | Number of ducts containing one or more strictures AND dilatations |
|  | Number of strictures with length < 3 mm |
|  | Number of strictures with length in range 3-10 mm |
|  | Number of strictures with length > 10 mm |
| Length of the pathology | Mean length of ducts (mm) |
|  | Median length of ducts (mm) |
|  | Maximum duct length (mm) |
|  | Sum of lengths of all ducts (mm) |
|  | Mean length of candidate strictures (mm) |
|  | Maximum length of candidate strictures (mm) |
|  | Total length of candidate strictures (mm) |
|  | Mean length of candidate dilatations (mm) |
|  | Maximum length of candidate dilatations (mm) |
|  | Total length of candidate dilatations (mm) |
|  | Total length of strictures and dilatations (mm) |
| Severity of the pathology | Mean absolute severity of candidate strictures |
|  | Maximum absolute severity of candidate strictures |
|  | Sum of absolute severity of candidate strictures |
|  | Mean relative severity of candidate strictures |
|  | Maximum relative severity of candidate strictures |
|  | Sum of relative severity of candidate strictures |
|  | Mean relative severity score for candidate strictures |
|  | Maximum relative severity score for candidate stricture |
|  | Sum of the relative severity score for candidate strictures |
|  | Total number of candidate dilatations |
|  | Mean absolute severity of candidate dilatations |
|  | Sum of absolute severity of candidate dilatations |
|  | Mean relative severity of candidate dilatations |
|  | Maximum relative severity of candidate dilatations |
|  | Sum of relative severity of candidate dilatations |
|  | Mean relative severity score for candidate dilatations |
|  | Maximum relative severity score for candidate dilatations |
|  | Sum of relative severity scores for candidate dilatations |
|  | Percentage of ducts containing one or more strictures OR dilatations |
|  | Percentage of ducts containing one or more strictures AND dilatations |
|  | Percentage of tree centreline which is abnormal (%) |
|  | Maximum absolute severity of candidate dilatations |
| Basic metrics of biliary tree | Median duct diameter (mm) |
|  | Minimum diameter of candidate stricture (mm) |
|  | Maximum diameter of dilatation |
|  | Percentage duct median diameters <1 mm |
|  | Percentage duct median diameters in range 1-3 mm |
|  | Percentage duct median diameters in range 3-5 mm |
|  | Percentage duct median diameters in range 5-7 mm |
|  | Percentage duct median diameters in range 7-9 mm |
|  | Percentage duct median diameters <9 mm |
|  | Percentage points along the centreline with diameter < 1 mm |
|  | Percentage points along the centreline with diameter in range 1-3 mm |
|  | Percentage points along the centreline with diameter in range 3-5 mm |
|  | Percentage points along the centreline with diameter in range 5-7 mm |
|  | Percentage points along the centreline with diameter in range 7-9 mm |
|  | Percentage points along the centreline with diameter in range < 9 mm |

Supplementary table 2: Correlations between imaging metrics and liver biochemistry

|  | **ALT** | *p* | **AST** | *p* | **GGT** | *P* | **total**  **bilirubin** | *p* | **IgG** | *p* | **Gamma**  **globulins** | *p* |
| --- | --- | --- | --- | --- | --- | --- | --- | --- | --- | --- | --- | --- |
| Time between diagnosis and MRI | -0.48 | *<0.001* | -0.5 | *<0.001* | -0.28 | *0.056* | -0.2 | *0.177* | -0.36 | 0.012 | -0.39 | *0.007* |
| **mpMRI metrics** | | | | | | | | | | | | |
| PDFF | -0.38 | *0.007* | -0.46 | *0.001* | -0.35 | *0.013* | -0.35 | *0.013* | -0.38 | 0.007 | -0.43 | *0.002* |
| cT1 | 0.52 | *<0.001* | 0.56 | *<0.001* | 0.5 | *<0.001* | 0.46 | *0.001* | 0.34 | 0.015 | 0.24 | *0.092* |
| **Biliary tree parameters (MRCP+ metrics)** | | | | | | | | | | | |  |
| Percentage of the ducts with median diameter ranging 1-3 mm | -0.08 | 0.592 | 0.05 | 0.733 | -0.05 | 0.729 | 0.01 | 0.943 | -0.31 | 0.032 | -0.15 | 0.289 |
| Percentage of the ducts with median diameter ranging 3-5 mm | *NS* |  | *NS* |  | *NS* |  | *NS* |  | *NS* |  | *NS* |  |
| Percentage of the ducts with median diameter ranging 5-7 mm | *NS* |  | 0.31 | *0.032* | 0.31 | *0.032* | *NS* |  | *NS* |  | *NS* |  |
| Percentage points along the bile duct centreline with diameter < 1 mm | 0.25 | 0.083 | 0.31 | 0.029 | 0.19 | 0.181 | 0.35 | 0.013 | -0.01 | 0.932 | 0.09 | 0.553 |
| Percentage points along the bile duct centreline with diameter ranging 1-3 mm | -0.01 | 0.92 | 0.07 | 0.641 | -0.02 | 0.872 | -0.08 | 0.587 | -0.22 | 0.123 | -0.08 | 0.605 |
| Percentage points along the bile duct centreline with diameter ranging 3-5 mm | *NS* |  | *NS* |  | 0.02 | *0.901* | 0.04 | *0.779* | *NS* |  | *NS* |  |
| Percentage points along the bile duct centreline with diameter ranging 7-9 mm | 0.29 | *0.04* | *NS* |  | 0.41 | *0.003* | 0.32 | *0.025* | 0.32 | 0.025 | 0.3 | *0.036* |
| Percentage points along the bile duct centreline with diameter < 9 mm | 0.3 | *0.038* | 0.32 | *0.023* | 0.37 | *0.008* | *NS* |  | 0.35 | 0.014 | *NS* |  |
| Total number of strictures | *NS* |  | *NS* |  | 0.31 | *0.031* | *NS* |  | *NS* |  | *NS* |  |
| Sum of absolute severity of strictures | *NS* |  | *NS* |  | 0.31 | *0.032* | *NS* |  | *NS* |  | *NS* |  |
| Sum of relative severity of strictures | *NS* |  | *NS* |  | 0.29 | *0.042* | *NS* |  | *NS* |  | *NS* |  |
| Total number of dilatations | *NS* |  | *NS* |  | 0.31 | *0.03* | *NS* |  | *NS* |  | *NS* |  |
| Total length of dilatations (mm) | 0.29 | *0.042* | *NS* |  | 0.43 | *0.002* | *NS* |  | *NS* |  | *NS* |  |
| Maximum dilatation diameter | *NS* |  | *NS* |  | 0.36 | *0.01* | 0.32 | *0.025* | *NS* |  | *NS* |  |
| Sum of relative severity of candidate dilatations | *NS* |  | *NS* |  | 0.28 | *0.049* | -0.03 | *0.855* | *NS* |  | *NS* |  |
| Number of ducts with strictures | *NS* |  | *NS* |  | 0.32 | *0.024* | *NS* |  | *NS* |  | *NS* |  |
| Number of ducts with dilatations | *NS* |  | *NS* |  | 0.34 | *0.015* | *NS* |  | *NS* |  | *NS* |  |
| Number of ducts with strictures/dilatations | *NS* |  | *NS* |  | 0.34 | *0.015* | *NS* |  | *NS* |  | *NS* |  |
| Total length of ducts with abnormalities (strictures and dilatations) (mm) | *NS* |  | *NS* |  | 0.32 | *0.024* | *NS* |  | *NS* |  | *NS* |  |

Supplementary table 3: Correlations between imaging metrics, liver biochemistry and histology

|  | **Fibrosis** | *p* | **Lobular**  **inflammation** | *p* | **Portal**  **inflammation** | *p* |
| --- | --- | --- | --- | --- | --- | --- |
| Time between diagnosis and MRI | *NS* |  | -0.34 | *0.016* | *NS* |  |
| **Liver biochemistry** | | | | | | |
| ALT | 0.44 | *0.001* | 0.36 | *0.012* | 0.36 | *0.011* |
| AST | 0.52 | *0.001* | 0.37 | *0.009* | 0.36 | *0.01* |
| GGT | 0.41 | *0.004* | *NS* |  | *NS* |  |
| total bilirubin | 0.39 | *0.005* | *NS* |  | *NS* |  |
| IgG | 0.32 | *0.026* | 0.39 | *0.005* | 0.52 | *0.0001* |
| Gamma globulins | *NS* |  | 0.32 | *0.026* | 0.5 | *0.0003* |
| **mpMRI metrics** | | | | | | |
| PDFF | -0.28 | *0.047* | -0.27 | *0.064* | *NS* |  |
| cT1 | 0.42 | *0.002* | 0.31 | *0.03* | 0.41 | *0.003* |
| **Biliary tree parameters (MRCP+ metrics)** | | | | | | |
| Percentage of the ducts with median diameter ranging 1-3 mm | -0.34 | *0.018* | *NS* |  | *NS* |  |
| Percentage of the ducts with median diameter ranging 3-5 mm | 0.35 | *0.013* | *NS* |  | *NS* |  |
| Percentage of the ducts with median diameter ranging 5-7 mm | *NS* |  | *NS* |  | *NS* |  |
| Percentage points along the bile duct centreline with diameter < 1 mm | *NS* |  | 0.31 | *0.029* | *NS* |  |
| Percentage points along the bile duct centreline with diameter ranging 1-3 mm | -0.31 | *0.031* | *NS* |  | *NS* |  |
| Percentage points along the bile duct centreline with diameter ranging 3-5 mm | 0.32 | *0.024* | *NS* |  | *NS* |  |
| Percentage points along the bile duct centreline with diameter ranging 7-9 mm | *NS* |  | *NS* |  | *NS* |  |
| Percentage points along the bile duct centreline with diameter < 9 mm | *NS* |  | *NS* |  | *NS* |  |
| Total number of strictures | 0.34 | *0.016* | *NS* |  | *NS* |  |
| Sum of absolute severity of strictures | *NS* |  | *NS* |  | *NS* |  |
| Sum of relative severity of strictures | *NS* |  | *NS* |  | *NS* |  |
| Total number of dilatations | *NS* |  | *NS* |  | *NS* |  |
| Total length of dilatations (mm) | *NS* |  | *NS* |  | *NS* |  |
| Maximum dilatation diameter | *NS* |  | *NS* |  | *NS* |  |
| Sum of relative severity of candidate dilatations | *NS* |  | *NS* |  | *NS* |  |
| Number of ducts with strictures | *NS* |  | *NS* |  | *NS* |  |
| Number of ducts with dilatations | *NS* |  | *NS* |  | *NS* |  |
| Number of ducts with strictures/dilatations | *NS* |  | *NS* |  | *NS* |  |
| Total length of ducts with abnormalities (strictures and dilatations) (mm) | *NS* |  | *NS* |  | *NS* |  |
